# Note from Dr. Gaston

**Published:** 1889-09

**Authors:** J. McFadden Gaston


HYDROPHOBIA.—NOTE FROM DR. GASTON.—In connection with the communication of Dr. J. D. Bradley, I would state that the facts repeated indicate that the case was undoubtedly hydrophobia.

While most cases of this affection are traceable to the bites of other animals, there are reasons to believe that it occurs in dogs and other inferior animals without such contact, and hence is of spontaneous origin.

The results of Pasteur's treatment of cases which have been bitten by dogs supposed to be rabid are not conclusive, but his experiments with attenuated virus by inoculation demonstrate that immunity is conferred upon dogs by such a process. It is therefore a legitimate inference that a similar proceeding with human beings would secure them against this affection. The antidotal curative properties of inoculation after the disease has been contracted is, to say the least, extremely doubtful. But the prophylactic virtues of inoculation ought to be available with the human race.

A thermometer used under the tongue of the subject of hydrophobia may be freed from all traces of the saliva by washing with a proper disinfectant, so that there could be no risk in using it afterward. But there is no instance reported within our knowledge in which this disease has been communicated by the saliva of a human being, even when bites have been inflicted.

J. McFADDEN GASTON, M. D.

